# USING LIFE REVIEW TO EXPLORE DEATH EXPERIENCES AND SELF-MANAGEMENT AMONG HEALTHY OLDER ADULTS IN TAIWAN

**DOI:** 10.1093/geroni/igae098.2394

**Published:** 2024-12-31

**Authors:** Hsin-Ying Tsou, Yu- Ten Pan, Tsuann Kuo

**Affiliations:** Chung Shan Medical University, Taichung, Taichung, Taiwan (Republic of China); Pingtung Ailiao Community Development Association, Pingtung, Pingtung, Taiwan (Republic of China); Chung Shan Medical University, Taichung, Taichung, Taiwan (Republic of China)

## Abstract

**Background:**

Discussing death is considered a culturally sensitive topic in Eastern societies, as it may evoke concerns related to inheritance, filial piety, and mortality. Avoiding conversation about death can result in family members guessing the wishes of the deceased and may cause disputes and conflicts within the family. Purpose This study aims to explore the changing process of understanding death and self-arrangement among 11 healthy older adults in Taiwan through the use of life review program.

**Method:**

This paper examines participation, dialogue, changes, and reflection in ten life review group sessions with 11 healthy older adults through participant observations and document text analysis. The sessions took place weekly for two hours between April to June in 2023.

**Result & discussion:**

The process to reflect deaths and their self-management included four stages. 1) The “resistance” stage: participants expressed uncertainty by stating “I don’t know how to talk about it”. 2) In “promotion” stage: participants were encouraged to share their experiences in managing funeral arrangements for family members, leading them to realize that “there is no standard answer for funerals, only respect for the deceased”. 3) During the “change” stage: participants were willing to make arrangements regarding properties, medical decisions, funeral arrangements of their own. 4) In the “sharing” stage: an act was performed to summarize the course and to recognize that everyone was unique.

**Conclusion:**

The life review group can effectively guide older adults to discuss their death experience and self-arrangement.

